# Exosome Biogenesis, Regulation, and Function in Viral Infection

**DOI:** 10.3390/v7092862

**Published:** 2015-09-17

**Authors:** Marta Alenquer, Maria João Amorim

**Affiliations:** Cell Biology of Viral Infection Lab, Instituto Gulbenkian de Ciência, Rua da Quinta Grande, 6, 2778-156 Oeiras, Portugal; malenquer@igc.gulbenkian.pt

**Keywords:** biological sciences, microbiology, virology, exosomes, endocytic pathways, immunity, mechanisms of viral spread

## Abstract

Exosomes are extracellular vesicles released upon fusion of multivesicular bodies (MVBs) with the cellular plasma membrane. They originate as intraluminal vesicles (ILVs) during the process of MVB formation. Exosomes were shown to contain selectively sorted functional proteins, lipids, and RNAs, mediating cell-to-cell communications and hence playing a role in the physiology of the healthy and diseased organism. Challenges in the field include the identification of mechanisms sustaining packaging of membrane-bound and soluble material to these vesicles and the understanding of the underlying processes directing MVBs for degradation or fusion with the plasma membrane. The investigation into the formation and roles of exosomes in viral infection is in its early years. Although still controversial, exosomes can, in principle, incorporate any functional factor, provided they have an appropriate sorting signal, and thus are prone to viral exploitation. This review initially focuses on the composition and biogenesis of exosomes. It then explores the regulatory mechanisms underlying their biogenesis. Exosomes are part of the endocytic system, which is tightly regulated and able to respond to several stimuli that lead to alterations in the composition of its sub-compartments. We discuss the current knowledge of how these changes affect exosomal release. We then summarize how different viruses exploit specific proteins of endocytic sub-compartments and speculate that it could interfere with exosome function, although no direct link between viral usage of the endocytic system and exosome release has yet been reported. Many recent reports have ascribed functions to exosomes released from cells infected with a variety of animal viruses, including viral spread, host immunity, and manipulation of the microenvironment, which are discussed. Given the ever-growing roles and importance of exosomes in viral infections, understanding what regulates their composition and levels, and defining their functions will ultimately provide additional insights into the virulence and persistence of infections.

## 1. Literature Review

Multivesicular bodies (MVBs) or late endosomes are components of the endocytic pathway that range from 250 to 1000 nm in diameter. Within MVBs are vesicles called intraluminal vesicles (ILVs) that range from 30 to 100 nm in diameter. MVBs can either be degraded or can fuse with the plasma membrane, releasing the ILVs into the extracellular space. The ILVs are called exosomes following their release from the MVB [1]. The formation of the ILVs within the MVB and the budding of enveloped virions share many features. Both processes require induction of membrane curvature, inclusion of specific cargo, and membrane fission for release. What is most striking is that evolutionarily unrelated viruses, with dramatically different genomes, have converged in their use of the host machinery for ILV formation to promote their own budding. Important human pathogens such as the human immunodeficiency virus (HIV), the Ebola virus, the rabies virus, and the herpes simplex virus 1 (HSV1) all have well-characterized strategies to hijack members of the endosomal sorting complexes required for the transport (ESCRT) pathway [2,3]. The ESCRT pathway is the best understood mechanism underlying ILV biogenesis [4]. However, there are viruses that manage to bud from cells via ESCRT-independent pathways. Classical examples include the influenza A virus (IAV), the severe acute respiratory syndrome Corona virus, alphaviruses like chikungunya, and pneumoviruses like respiratory syncytial virus (RSV). To add another layer of complexity, regardless of whether ESCRT is involved in viral budding, recent publications have reported roles of exosomes in viral infection far beyond assisting with the assembly of enveloped viruses. In this review we explain how exosomes are part of the endocytic pathway and how their biogenesis is regulated. We also discuss the different strategies used by viruses to subvert these regulatory mechanisms for their own profit. Finally, we explore the roles of exosomes in viral spread, immune regulation and tumor development.

## 2. Apoptotic Bodies, Microvesicles, and Exosomes

**Figure 1:**
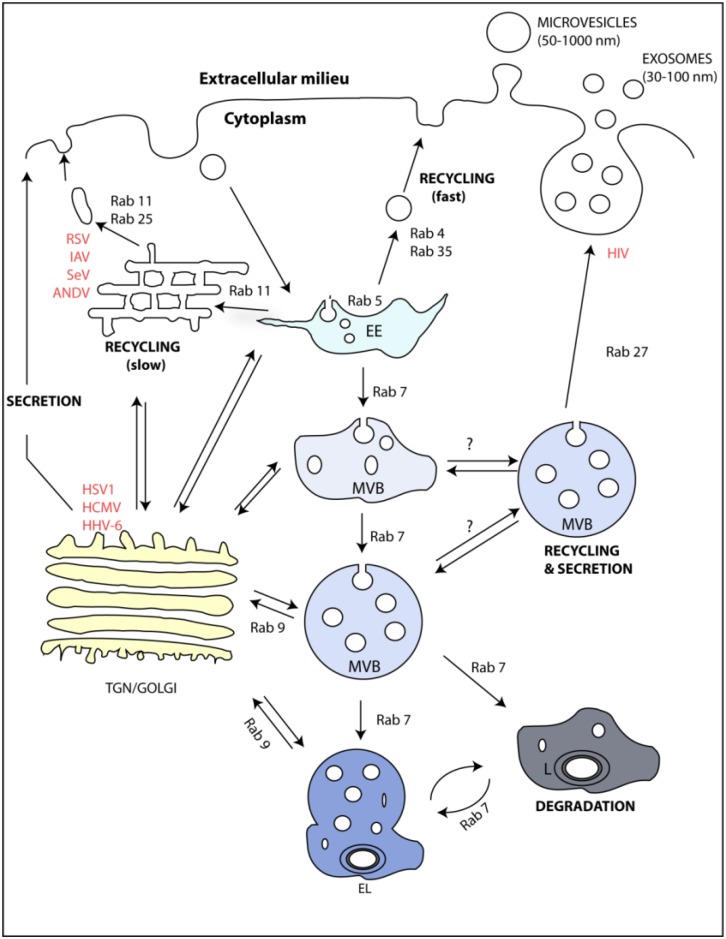
The endocytic and secretory pathways. Cargo binds to the plasma membrane, is endocytosed by a plethora of processes and, independently of the entry route, is transported to early endosomes (EE). From this sub-compartment, cargo is sorted to one of three destinations: recycling, degradation, or secretion. These routes require maturation of the EE into recycling endosomes or multivesicular bodies (MVBs), which can either fuse with lysosomes (L) to generate endolysosomes (EL) or with the plasma membrane to release intraluminal vesicles to the milieu as exosomes. The membranes of the sub-compartments of the endocytic pathway have different compositions. Specific members of the Rab GTPase family, for example, are differentially enriched in each sub-compartment: Rab5 is enriched in EE; Rab7 in MVBs; Rab11, Rab25, Rab4, and Rab35 in the slow and rapid recycling routes; and Rab27a/b in MVBs. Rab9 is present in vesicles destined for retrograde transport to the trans-Golgi network (TGN). In uninfected cells, interfering with these Rabs affects exosome release. Many viruses use these Rabs in diverse steps of the viral life cycle, although whether this usage impacts in exosomal release has not been investigated. For example, at late stages of infection, viruses such as IAV, RSV, Sendai virus (SeV), and Andes virus (ANDV) were shown to hijack Rab11 vesicles to transport their progeny RNA to the cell surface. HIV, HSV1, and human cytomegalovirus (HCMV) were shown to require Rab27a/b vesicles for assembly. Human herpes 6 (HHV-6) virions were shown to be secreted upon fusion of MVB with the plasma membrane, together with exosomes.

The cytoplasm of the eukaryotic cell contains several well-described compartments (trans-Golgi network (TGN), mitochondria, peroxisomes, endoplasmic reticulum, *etc.*), each performing specific and, in some cases, overlapping functions. Transport of materials (metabolites, lipids, carbohydrates, and proteins) between organelles has been described to be mediated by vesicles of about 60–100 nm diameter, moving in a densely populated microenvironment [5,6] (reviewed in [7]). Vesicles are also used for intercellular communication, to receive and send signals. These have different sizes, distinct origins, and are formed using a multitude of mechanisms. It has long been known that apoptotic cells shed large vesicles of about 500–2000 nm in diameter that can be taken up by phagocytic or antigen-presenting cells, the latter being important regulators of the immune system [8]. Subsequently, it was shown that healthy cells also release vesicles able to mediate intercellular communication. Such vesicles are of two types: (1) microvesicles, 50–1000 nm in diameter, are formed at the cell surface (Figure 1); and (2) exosomes, 30–100 nm in diameter, are initially formed as ILVs inside the MVB (Figure 1), and are released upon fusion of the MVB with the plasma membrane [9]. Exosomes, conserved structures formed in every cell type [10], were identified over 25 years ago during the differentiation of erythrocytes [9,11]. Most bodily fluids contain exosomes [12]. Their contents have been shown to change in various diseases including viral infections, neurodegenerative diseases (prions, Alzheimer, Huntington disease), and cancer, and hence exosomes are being intensively investigated as a source of novel biomarkers [13]. In recent years, a plethora of reports and reviews has explored several functions of exosomes in mediating intercellular communication, immune system functions, development and differentiation, neuronal function, cell signaling, regeneration [1,14,15,16,17,18,19,20,21], and several steps in viral replication, the latter topic explored in this review.

## 3. Endocytic Pathway and Exosomes

The endocytic pathway is a convoluted web of interconnected sub-compartments with distinct cell localization, lipid and protein composition, and pH, which operates as follows: cells internalize ligands by endocytosis concomitantly with membrane proteins and lipids [22,23]. Irrespectively of the route of entry, internalized material is delivered to early endosomes and sorted to at least three possible destinations, as shown in Figure 1. The internalized material can be sent for degradation through maturation into MVBs and fusion with lysosomes, which are acidic compartments containing hydrolytic enzymes able to digest complex macromolecules [24]. Alternatively, cargo can be re-routed for recycling or secretion. Recycling processes are categorized into a quicker and a slower pathway, according to the time that proteins (and lipids) take from internalization to exposure back at the cell surface (or release to the extracellular media in case of luminal soluble factors), which is described in more detail below [25]. Secretion of exosomes requires maturation of early endosomes into MVBs (a process reviewed in [26]), with concomitant formation of ILVs, and fusion of MVBs with the cell surface to release exosomes. At any point, material can be further internalized to the TGN and integrated in canonical secretory pathways [26,27].

No underlying mechanism has yet been reported to differentiate MVB formation for degradation or fusion with the cell membrane. There are, however, reports suggesting the existence of subpopulations of MVBs, depending on their fate. Using a biotinylated derivative of the cholesterol-binding toxin perfringolysin O from *Clostridium perfringens* to localize cholesterol at the subcellular level, Möbius *et al*. identified two types of MVBs of similar morphology, one cholesterol-rich that was destined for secretion, and the other cholesterol-poor, which was destined for degradation [28]. Conversely, two sub-populations of MVBs differing in the presence or absence of lysobisphospatidic acid have been described. For example, EGF and its receptor (EGFR) are only present in MVBs negative for lysobisphospatidic acid [29]. This supports the previously described role of phosphatidyl inositol (PI) in the formation of MVBs containing EGFR [30], but not lysobisphospatidic acid [31]. Another study showed that a full-length p75 receptor was only released from sympathetic neurons and PC12 cells upon KCl-mediated cell depolarization. Under this stimulus, p75-containing MVBs escaped endolysosomes and degradation [32]. Whether this reflects distinct steps in MVB trafficking or even distinct mechanisms in MVB formation that mark them for degradation or fusion with the membrane is still unclear.

ILV formation is characterized by inward budding of membranes, a process that starts in early endosomes but greatly augments as endosomes mature [26]. Evidence indicates that exosomes correspond to secreted ILVs of MVBs. Relative to the composition of the cytoplasm, exosomes are enriched in components such as lipids, RNAs, and proteins. Lipids include cholesterol, sphingomyelin, glycosphingolipids, and phosphatidylcholine with saturated fatty acids [33,34,35]. Enriched RNAs are specific miRNAs, non-coding RNAs, tRNAs, rRNAs, and mRNAs [36,37,38,39]. Finally, proteins found in higher concentration than in the cytosol include specific factors of the immune system, those of the ESCRT apparatus, those involved in trafficking, and lipid-rafts residents. The latter are, for example, cytokines, tetraspanins, Major Histocompability Complex (MHC) class I and II molecules, glycosylphosphatidylinositol-anchored proteins, Rabs, SNARES, and flotillin [1,20,38,40,41,42]. Importantly for the context of this review, cells infected with viruses were shown to release exosomes containing viral proteins and RNAs [43,44,45,46,47,48,49,50] (reviewed in [49]). Some viral proteins, including the HIV Nef, seem to have exosomal localization signals [46,51]. However, in the majority of cases, it is unclear whether the inclusion of viral components in exosomes results from direct sorting or from hijacking the machinery for exosome biogenesis, trafficking, and/or release. Nevertheless, the specific composition of exosomes derived from cells, regardless of their infectious state, suggests that soluble and membrane-bound cargo are selectively incorporated into ILVs [1,20,36,37,38,39,40,41,42].

The formation of ILVs is accomplished by several molecular mechanisms. Proteins belonging to the ESCRT family are, by far, the best characterized and have been extensively reviewed [1,12,15,52,53,54]. There are, however, ubiquitin- and ESCRT-independent pathways [4], including the oligomerization of the tetraspanin complexes [55], the sphingomyelinase pathway that catalyzes ceramide synthesis [56], or phospholipase D2 and ADP rybosylation factor-6-mediated ILV budding [57]. One way for viral modulation of exosome release is by directly interfering with the machinery involved in exosome biogenesis; this has been reviewed elsewhere for the ESCRT machinery [3]. However, in principle, anything that hinders MVB formation might impact, even if indirectly, on exosome biogenesis. For a clearer exploration of the latter hypothesis, we will briefly summarize some regulatory mechanisms operating in the endocytic pathway, the system in charge of exosome formation.

## 4. Rabs as Regulators of the Endocytic Pathway and Exosome Formation

There has been much debate on whether vesicles that deliver material from a donor compartment to a specific acceptor sub-organelle contain information on where to go [58,59]. In the 1990s, it was found that different compartments, and the vesicles they produced, were populated by distinct Rab GTPases. The Rab GTPases belong to a large family of highly conserved proteins with 60 members, which were discovered to regulate vesicular trafficking in eukaryotes [60]. The description of the endocytic pathway in Figure 1 can be explained with a set of Rabs as follows: after endocytosis, sorting in Rab5-positive early endosomes [61] delivers cargo to return to the plasma membrane along fast (Rab4, Rab35) [62] or slow (Rab11a, Rab11b, Rab25) [63,64] recycling processes. Alternatively, Rab5 endosomes acquire Rab7 and release Rab5 by a process called endosome maturation [65,66]. Rab7-containing endosomes sort material to ILV, decrease pH, and acquire hydrolytic proteases able to degrade their internal contents [67], or instead acquire Rab27a/b and fuse with the plasma membrane-releasing exosomes [68]. At any point, vesicles might acquire Rab9 to enter a retrograde transport to the TGN [66].

In recent years, it has been shown that interfering with the levels and activation of Rab GTPases influences exosome release. Depending on the cell type, Rab11, Rab27, Rab35, Rab5, and Rab7 were all implicated in the release of vesicles. Rab5 overexpression was shown to inhibit progression of endocytosed material from early endosomes, impacting negatively on exosomal release of markers such as syndecan, CD63, and Alix, and this reduction was rescued by the overexpression of Rab7 [69]. In agreement, Rab7 depletion severely impaired exosomal release of the same factors [69]. The lack of a functional active Rab11 reduced the secretion of exosomes in the erythroleukemia cell line K568, *Drosophila* S2 cells, and retinal epithelial cells, as evaluated by the following exosomal proxies: transferrin and HSC-70, Wingless and Evi, or flotillin and anthrax-toxin, respectively [70,71,72,73].Rab35 was identified in a screen performed in oligodendroglial cancer and primary cells to analyze phospholipase D2-containing exosomes [23]. Rab27 has been found to facilitate the release of the exosomal markers MHC II, CD63, and CD81 in many cancer types, including HeLa cells [68,74,75]. Interestingly, Rab27 and Rab35 did not influence the release of Wingless in *Drosophila* S2 cells [71] and Rab27 did not affect the extracellular levels of flotillin or of anthrax toxin in retinal epithelial cells [70]. The roles of these Rabs in the endocytic pathway allowed for speculation that there might be extracellular vesicles derived from different routes such as recycling and MVB, but this awaits confirmation [15].

In conclusion, altering the levels of any of the referred Rabs has the potential to interfere with the progression of cargo at specific endocytic locations. Consistently, it has been shown that switching off Rab5 and repopulating the endosome with other Rabs is a prerequisite for the maturation of early endosomes into other types of endosomes [61,76]. Conversion to Rab7 forms MVBs and endolysosomes. MVB acquisition of Rab27 is, in some cases, required for exosome release [67,68]. Additionally, exchanging Rab5 for Rab11 or Rab25 allows progression from early endosomes to the slow recycling route and exchanging Rab5 for Rab35 or Rab4 allows progression from early endosomes to the fast recycling route [22].

## 5. Viruses and Rab GTPases Involved in Exosome Formation

Many viruses were shown to use the Rabs mentioned above to assist several steps of their replication. There is no evidence yet relating the viral usage of these proteins with exosome biogenesis and function. The picture of the viral usage of endocytic proteins has been built during years of research, making of viruses excellent tools to understand the crosstalk between different endocytic sub-compartments. In this sense, we provide an overview of identified interactions between viruses and endocytic proteins that regulate exosome biogenesis. Examples of viruses using these Rabs are identified in red in Figure 1 and in Table 1.

The *Orthomyxovirus* IAV, the *Paramyxoviruses* SeV and RSV, and the *Bunyavirus* ANDV all share negative strand RNA genomes, infect the lung epithelia, and use Rab11a pathway in their infectious cycle [73,77,78,79,80,81,82]. In the case of SeV, RSV, and IAV, progeny RNA (in the form of viral ribonucleoproteins, vRNPs) attach, facing the cytoplasm, to Rab11 vesicles as a way to facilitate their transport to the apical side of the plasma membrane. For these three viruses and conversely to ANDV, viral interaction with the recycling endosome occurs via activated (GTP-bound) Rab11a [73,77,78,79,80,81,82].

The impact of Rab11 viral hijacking in the recruitment and activation of other Rabs and exosome biogenesis has not been investigated. However, as mentioned above, interfering with Rab11 levels can inhibit or promote the release of exosomes containing transferrin, HSC-70, flotillin, and anthrax toxin [70,71,72,73]. During IAV infection, the total levels of Rab11 remain fairly constant, but it is possible that the amount of the activated form suffers viral-induced fluctuations. Mechanistically, in uninfected cells, vesicular transport is promoted by binding of molecular motors to activated Rab11. Amongst many of the Rab11 effectors reported [83], the members of the Rab11-interacting family proteins (FIPs) have been well characterized in facilitating vesicular movement [84]. Reduction in the levels of some FIPs was reported to interfere with sorting of recycling vesicles [85,86]. For IAV, our unpublished results suggest that the vRNP hijacking of the Rab11 pathway “slows down” recycling efficiency. This system could then be used to assess the effects of impairing Rab11 in exosome biogenesis. The effects in recycling efficiency might differ for RSV, as Utley *et al.* [82] have shown that several members of the recycling machinery were required for RSV vRNP transport. In the case of ANDV, viral replication and assembly is thought to occur in the lumen of a membranous delimited sub-compartment, followed by budding to the cytoplasm and transport to the periphery. Rab11 depletion was shown to reduce over 10-fold the levels of produced virions [81]. In this case, the viral structural protein N was shown to co-localize mainly with the GDP-bound Rab11 near the TGN, at a perinuclear location, although it has not yet been addressed whether ANDV inhibits Rab11 activation or affects MVB formation and exosome release [81]. Such analysis has also not been performed for RSV and SeV.

**Table 1:** Established associations between viruses and sub-compartments from the endocytic system. Viral and host proteins involved in these associations are shown. The status of Rab11 required for the virus association is provided as activated (GTP-bound) or inactivated (GDP-bound). References where alterations in exosome biogenesis were identified are provided, if available. Although ESCRT members participate in exosome biogenesis and have clear roles in viral infection, we have not mentioned their viral usage in this manuscript. Readers are directed to other reviews on the topic [3,87].

Virus	Interaction with Endocytic Sub-compartment	Host Protein	Viral Protein	Ref	Alteration in Exosome Biogenesis	Ref.
IAV	Recycling endosome	Rab11 (GTP)	vRNP (possibly PB2)	[77,78,79,80,88]	No evidence	
SeV	Recycling endosome	Rab11 (GTP)	vRNP	[89]	No evidence	
RSV	Recycling endosome	Rab11 (GTP)	No evidence	[82]	No evidence	
ANDV	Recycling endosome	Rab11 (GDP)	N	[81]	No evidence	
HIV	MVB	Rab27	Pr55^Gag^	[90]	HIV Nef protein increases the production of exosomes and is secreted in exosomes	[46,91]
HCMV	MVB	Rab27	No evidence	[92]	No evidence	
HSV1	MVB	Rab27	GHSV-UL46	[93]	Glycoprotein B Diverts HLA-DR into the Exosome Pathway	[94]
HHV-6		CD63 (association not proven)	Virions inside MVBs shown by electron microscopy	[95]	No evidence	

Rab27a regulates secretion of lysosome-related organelles, including MVBs [68,74]. It was reported that Rab27a levels increased in HCMV-infected cells [92], a DNA virus member of the *Betaherpesvirinae* subfamily. This Rab was found in association with viral envelopes of HCMV in an undefined compartment related to the TGN or in vesicles being transported between the TGN and endosomes [92]. The molecular mechanisms leading to increased levels of Rab27a in HCMV infection are still unclear, as well as their interference with exosome release. However, given the positive role of Rab27a in extracellular MHC II-containing exosomes and the regulation of immune responses in glial cells, this role of Rab27a in HCMV infection should be further evaluated [92]. Another virus that hijacks Rab27a to promote assembly is HIV (Figure 1), by a mechanism that is described below [90]. HIV also uses the ESCRT pathway to facilitate budding [3] and additionally increases the transcription of genes involved in MVB formation and exosome release [46]. This viral interference with many different steps of MVB/exosome formation suggests a high dependency on the host ILV machinery. In conclusion, much work has been done in understanding how different Rabs regulate particular steps in the life cycle of specific viruses. How the usage of various Rabs impacts the overall regulation of the endocytic pathway, including exosome release and their still controversial associated functions, remains to be evaluated.

## 6. Other Regulators of the Endocytic Pathway

**Figure 2:**
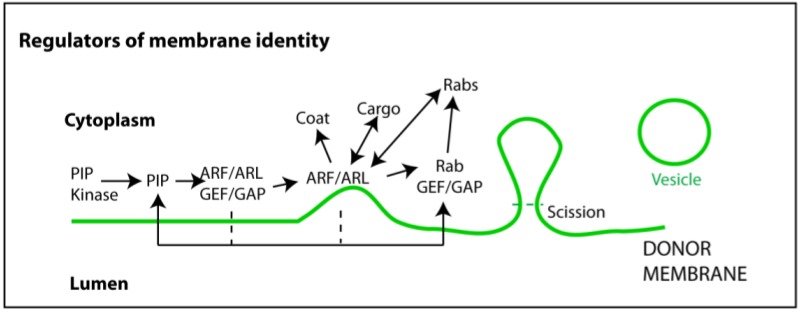
Regulators of membrane identity. Membrane composition is important to maintain the integrity of endocytic process. Phosphoinositide (PI) kinases (and phosphatases) ensure the levels of specific PIPs in distinct membranes. These operate as docking platforms for guanine exchange and activator factors (GEFs and GAPs) able to recruit and turn on/off GTPases. GTPases involved in membrane integrity and vesicular biogenesis are mainly of two kinds: ADP ribosylation factors (ARFs) and ARF-like proteins (ARLs); and Rabs. ARFs and ARLs are involved in early steps of vesicular biogenesis such as recruiting coating proteins and cargo, membrane curvature, and neck formation. Rabs operate at later stages by recruiting effectors such as molecular motors, which are able to generate pulling forces and move released vesicles. Vesicle scission and release are mediated by highly specialized proteins that recognize, encircle, and cut the membrane neck, using GTP or ATP hydrolysis to drive the reaction.

As mentioned above, it was shown by many independent groups and using several models that specific Rabs interfere with exosome release. However, Rabs are themselves subjected to tight regulation and are not the only factors controlling membrane identity [96,97]. Figure 2 depicts the complexity behind the composition of each sub-compartment membrane, with many factors required to create the correct microenvironment. Of these, seven phosphoinositide phosphates (PIP) are crucial to recruit specific Rabs, albeit indirectly (for a comprehensive review on PIP chemistry and biology please refer to [98,99]). These phospholipid derivatives are enriched in specific cellular membranes. For example, the plasma membrane contains mostly phosphoinositol-4,5-bisphosphate (PI(4,5)P_2_) and phosphoinositol-3,4,5-triphosphate (PI(3,4,5)P_3_), whilst the membranes of the endocytic pathway are decorated with phosphoinositol-3-phosphate (PI3P). During maturation of endosomes, MVBs acquire phosphoinositol-3,5-bisphosphate (PI(3,5)P_2_), a form that becomes prevalent in lysosomes [99]. The PIP isoforms are recruited by kinases and phosphatases that also occupy precisely defined sub-compartments [99]. Viruses have been shown to alter this PIP equilibrium and by doing so alter the content of Rabs involved in exosome formation. For example, phosphatidylinositol 4-phosphate (PI4P) was shown to be recruited by *Flavivirus* and *Picornavirus* to sites near the endoplasmic reticulum. Mechanistically, such enrichment was shown to be mediated by specific phoshoinositide (PI) kinases [100,101], one of them able to recruit Rab11 to the Golgi [102]. Another recent paper explored the mechanisms of HIV-1 particles’ assembly at the plasma membrane, a process that occurs in micro-domains enriched in PI(4,5)P_2_ and the viral protein Pr55^Gag^. It was found that in T cells, Rab27a (and some of its effectors) boosted PI(4,5)P_2_ production by delivering the MVB-associated kinase PI4KIIα to the cell surface [90]. Rab27a was also implicated in exosome release in T cells; therefore, these HIV and exosome release could, in principle, be intertwined, although such relation has not been investigated yet.

Other factors that control Rab delivery to specific membranes include the proteins that directly activate/deactivate them. Being GTPases, Rabs suffer cycles of GTP/GDP binding, which are a function of the protein families’ Rab guanine exchange factors (GEF) and guanine-activating proteins (GAP), respectively (Figure 2) (reviewed in [58,59,96,103,104,105]). There is also a clear cross-talk between Rab proteins and other regulators of vesicular trafficking called ADP-ribosylation factors (ARFs) [106,107]. ARFs are also GTPases, recruited by GEFs and GAPs that reside in specific membranes according to their PIP composition. The complete picture of the regulators controlling the location and levels of the Rabs mentioned above is far from complete and these factors have not been explored in the context of viral infection. However, it is widely accepted that cells react to stimuli to adjust the distribution and levels of intracellular PIPs as well as their cycles of degradation/secretion/recycling [29]. All the above considerations raise important questions concerning the interplay between regulators of the endocytic process, exosome release and viral usurpation of specific steps in the endocytic pathway. First, can viral modulation of any of these regulators alter the content and levels of released exosomes? And second, would control of the levels and composition of secreted exosomes have associated functions? These are interesting questions that deserve more attention in the near future, especially given the ever-growing functions of exosomes in viral infections.

## 7. Other Functions of Exosomes in Viral Infections

### 7.1. Spread of Viral Infection

In normal circumstances, cells show remarkable processes to communicate with the exterior, including the release of exosomes. As mentioned throughout this review, exosomes can transfer functional proteins, lipids, and distinctive sets of RNAs from cell to cell in homeostatic conditions [36,38,39]. In the last decades, exosomes were also shown to facilitate cell-to-cell transport of disease-related proteins involved in neurodegenerative disorders, such as prions [108] and beta-amyloid peptides [109]. This machinery could, in principle, also contribute to viral spread. For this to happen, two prerequisites would be necessary. First, viral RNA and proteins would need to access ILV. Indeed, vesicular stomatitis virus (VSV), dengue virus (and other *Flavivirus* members), and hepatitis C virus (HCV) components were found in these sub-compartments [110,111]. A relevant question is: how are soluble viral RNAs sorted into ILV? RNA incorporation into exosomes has been suggested to operate via a selective and conserved mechanism linked to the lipid content of vesicles. For a comprehensive review on specific mechanisms, readers are directed to [19]. Nevertheless, the contribution of RNA-binding proteins has been recognized. The hnRNPA2B1 RNA binding protein, for example, was shown to bind to a four triplet motif on miRNAs and transport them into exosomes [112]. In the case of viral transmembrane proteins, the most common mechanism of targeting molecules to ILVs is via ubiquitination and recruitment of the ESCRT machinery [4,53,54]. For HCV, it was shown that the ESCRT component Hrs is critical for release of nucleocapsid [113], and for hepatitis A virus (HAV) the ESCRT protein VPS4B and the accessory proteins Alix [114] were deemed crucial for ILV budding.

Second, exosomes would need to enter a recipient cell and release their infectious content into the cytoplasm. This mechanism was recently reported for HCV [113], where exosomes derived from infected human hepatoma cells containing full-length viral RNA, along with core and envelope proteins [115], were shown to be infectious and a major route of transmission. Interestingly, the non-enveloped virus HAV has been reported to acquire a host-derived membrane for cell-to-cell transmission and the virus is still able to replicate in the recipient cell. The exosomes containing HCV RNA and exosome-like vesicles containing whole HAV capsids were less susceptible to antibody neutralization, and consequently this transmission mechanism has been reported to operate as an immune evasion strategy [113,114,116]. Upon reaching their destinations, exosomes containing viral proteins and RNA would only be infectious provided they could enter target cells and reach the cytoplasm. Exosomes containing VSV and other *Flaviviruses* seem to enter the cell by being taken up by the endocytic pathway [110,111]. These two viruses were shown to escape late endosomes and reach the cytosol by a “back-fusion” process, a phenomenon by which ILVs fuse with the external late endocytic membranes [117]. Alternatively, exosomes containing viral antigens can induce signaling cascades at the surface upon binding their receptors to control host immune responses [118]. Research on exosome-mediated viral spread is still very limited; however, exosome modulation of the immune responses has been explored in some detail and will be discussed next.

### 7.2. Modulation of Immunity

One of the main functions assigned to exosomes is the mediation of intercellular communication during innate and adaptive immune responses. In fact, many different cells of the immune system, including dendritic cells and B and T lymphocytes, have been shown to release exosome vesicles with immune modulatory properties. These exosomes can be found in bodily fluids (reviewed in [119,120,121]).

In 1996, Raposo *et al*. demonstrated that B lymphocytes infected with Epstein-Barr virus (EBV), a human gammaherpesvirus associated with a variety of lymphoblastoid and epithelial cancers, released exosomes containing MHC II molecules, and that these vesicles were capable of activating specific CD4^+^ T cell clones *in vitro* [122]. Two years later, Zitvogel *et al*. published a study showing that exosomes released by dendritic cells had the ability to suppress the growth of tumors *in vivo*. This led to the interpretation that exosomes could be used as therapeutic agents modulating immune responses [123].

Subsequent studies with EBV-infected lymphoblastoid and nasopharyngeal carcinoma cells have shown that the exosomes secreted by these cells also harbor EBV-encoded latent phase mRNAs [124], proteins [43,45,125,126,127], and mature miRNAs [128,129]. Accumulating evidence suggests that these exosomes exert immune inhibitory effects on tumor-infiltrating lymphocytes. Latent membrane protein 1 (LMP1), the major viral oncogene expressed in most EBV-associated tumors, has been detected in exosomes and was shown to inhibit immune response, namely T lymphocyte activation and proliferation, NK cytotoxicity, and the ability of cells to produce interferon gamma [43,125,126]. Exosomes secreted by EBV-infected nasopharyngeal carcinoma cells also contained high amounts of the immunoregulator protein galectin-9, which is able to induce apoptosis of EBV-specific CD4^+^ T cells [126,130]. EBV miRNAs present in the exosomes are internalized by DC where they downregulate specific immunoregulatory genes [129]. In contrast, exosomes released from EBV-infected B lymphocytes were found to exert a stimulatory effect on non-infected B lymphocytes, driving their proliferation, class-switch recombination, and differentiation into plasmablast-like cells [131].

It was recently reported that exosomes also regulate innate immunity. This was illustrated with the identification of important innate immune effectors (IFI16, caspase-1, interleukin 1b (IL-1b), IL-18, and IL-33) in exosomes released from EBV-infected cells. Such a strategy removes these effectors from infected cells to reduce innate immunity activation [132]. Another interesting approach was proposed for HSV1 infection. In this case, cells export the innate immune sensor STING (stimulator of IFN genes), viral miRNAs, and mRNAs through exosomes that are delivered to uninfected cells [118]. The functional significance of this strategy is still not clear, but the fact that some miRNAs are able to suppress reactivation of latent virus suggests that, in specific circumstances, HSV1 has evolved mechanisms to restrict, rather than expand, the spread of infection. Exosomes harboring HCV RNA are transferred from infected cells to non-permissive plasmacytoid DCs, where viral RNA can trigger a type I IFN response [116].

It is clear that in viral infections, exosomes play a dual role in the modulation of the immune system, both serving as a host program to induce innate and adaptive immunity and as a viral strategy to evade those same responses.

### 7.3. Manipulation of Microenvironment

Viral infection is thought to be responsible for 10% to 15% of all human cancers, which makes the understanding of how pathogens modulate host cell functions during their transformation program seminal, both from a scientific and a clinical perspective [133]. Seven human tumor viruses have been identified, human papillomavirus (HPV), Merkel cell polyomavirus (MCV), HCV and hepatitis B virus (HBV), the members of the herpes family EBV and Kaposi’s sarcoma associated herpesvirus (KSHV), and the *Retrovirus* human T-lymphotropic virus-1. HIV is also tumorigenic, although indirectly, since the decrease of host immunity it provokes allows cell transformation, mostly by other viruses such as KSHV.

It is now well established that tumor cells secrete exosomes [18,35,134], but the cancer types in which exosomes are quantitatively, qualitatively, and functionally different from healthy tissues are incompletely characterized. Many studies reported differential composition of exosomes in healthy organisms *versus* those infected with several tumor viruses [127,131,135,136,137,138,139,140,141,142]. Nasopharyngeal carcinoma cells infected with EBV produced exosomes containing LMP1 [124,126]. These exosomes were shown to be taken up by neighboring cells [143] and the transcription profiles of the recipient cell were subsequently altered [128,143]. The mechanism underlying transcriptional alterations occurred via LMP1-mediated increase of EGFR release by exosomes that lead to activation of ERK and PI3K/Akt pathways in epithelial, endothelial, and fibroblast cells [128]. ERK and PI3K/Akt are renowned factors able to promote cell growth and migration. A specific miRNA composition has been found in tumor viruses’ derived exosomes [38,48,113,134,138,139,140,141,142]. For example, there are several types of HPV—some tumorigenic, each associated with a different miRNA exosomal profile [139,140,141]. In the case of exosomes isolated from cancer patients infected with tumorigenic HPV, the miRNA content was enriched for species controlling cell proliferation, senescence, and apoptosis. The exosomal miRNA compositions were dependent on the expression of the viral oncogene E6/E7, suggesting that this is one mechanism by which the oncogene contributes to the growth of HPV-positive cancer cells [142].

Regardless of the infection status, the significance of exosomes in tumor development was demonstrated in breast cancer, where normal cells became immortalized when incubated with exosomes derived from tumor cells [134]. Another example was the injection of Rab27a depleted breast carcinoma cell lines in immunocompetent mice. The lack of Rab27a was associated with reduced release of exosomes [68] and poor tumor development when compared to cells containing Rab27a, where tumor progression was normal and formed metastasis [74].

One of the major difficulties clinicians face is the lack of efficient methods to diagnose tumors, especially in early stages. In the case of tumors that result from viral infection, the identification of biomarkers for poor prognosis of infection would greatly benefit patients. Research needs to be done to identify clear populations of exosomes involved in infection and tumors, and to clearly define functions associated to exosomes in both conditions. These topics warrant investigation, as exosomes show great promise as biomarkers for cancer and/or infection, as therapeutic agents, and have the additional advantage of being accessible without the use of invasive techniques.

## 8. Future Perspectives

The mechanisms regulating the levels, content, and function of exosomes in viral infection remain poorly characterized. There are four areas that need detailed investigation.

First, it is unclear how the endocytic system controls the percentage of MVBs that fuse with the plasma membrane. We hypothesize that signaling events regulate the levels of activated Rabs at each step of the endocytic pathway, and that this fluctuation allows for an adjustment of the levels of biomolecules sent for degradation and/or secretion. Viruses provide excellent tools to better answer these questions as many efforts have been made to understand how each virus modifies distinct endocytic compartments. The next challenge is to better understand how these changes affect the endocytic process, including exosome release.

Second, little is known about the biological processes sorting material to ILVs of MVBs. The inclusion of viral proteins and RNAs in exosomes offers a unique system to identify signals and sorting mechanisms into ILVs.

Third, the functional relevance of exosomes in infection and disease remains incompletely characterized. Although exosome modulation of adaptive immunity has been extensively researched, its role in innate immunity remains largely unexplored. The recent identification of RNA transfer that might affect host gene expression has been an important discovery that has renewed interest in this field. The role of exosomes in viral spread is far less explored, namely its overall contribution to viral infection. Interestingly, using exosomes could be a means to mitigate exposure of viral antigens and operate as an immune evasion strategy. Clearly a lot has to be done in identifying viruses able to use this pathway and the mechanisms leading to the inclusion of viral proteins/RNA/capsids in ILVs—this also feeds into question two.

Finally, exosomes and many viruses share size, shape, and molecular characteristics. Technical improvements in methods to separate and obtain pure exosomal fractions will facilitate the understanding of their role in infection, namely in immune activation, viral spread, and persistence.
